# Identification of complex III, NQR, and SDH as primary bioenergetic enzymes during the stationary phase of *Pseudomonas aeruginosa* cultured in urine-like conditions

**DOI:** 10.3389/fmicb.2024.1347466

**Published:** 2024-02-21

**Authors:** Yuyao Hu, Ming Yuan, Alexander Julian, Karina Tuz, Oscar Juárez

**Affiliations:** Department of Biological Sciences, Illinois Institute of Technology, Chicago, IL, United States

**Keywords:** *Pseudomonas aeruginosa*, aerobic metabolism, respiratory chain, NQR, bc1 complex, SDH, urinary tract infection, metabolic activation

## Abstract

*Pseudomonas aeruginosa* is a common cause of urinary tract infections by strains that are often multidrug resistant, representing a major challenge to the world’s health care system. This microorganism has a highly adaptable metabolism that allows it to colonize many environments, including the urinary tract. In this work, we have characterized the metabolic strategies used by stationary phase *P. aeruginosa* cells cultivated in urine-like media to understand the adaptations used by this microorganism to survive and produce disease. Our proteomics results show that cells rely on the Entner-Duodoroff pathway, pentose phosphate pathway, the Krebs cycle/ glyoxylate shunt and the aerobic oxidative phosphorylation to survive in urine-like media and other conditions. A deep characterization of the oxidative phosphorylation showed that the respiratory rate of stationary phase cells is increased 3–4 times compared to cells in the logarithmic phase of growth, indicating that the aerobic metabolism plays critical roles in the stationary phase of cells grown in urine like media. Moreover, the data show that respiratory complex III, succinate dehydrogenase and the NADH dehydrogenase NQR have important functions and could be used as targets to develop new antibiotics against this bacterium.

## 1 Introduction

*Pseudomonas aeruginosa* is a Gram-negative opportunistic pathogen, ubiquitously distributed in natural and clinical environments (Bodey et al., 1983; Wiehlmann et al., 2007; Selezska et al., 2012). This bacterium relies on its highly flexible metabolism to survive under diverse and often harsh environmental conditions, which include antibiotic exposure (Livermore, 2002; Laborda et al., 2021). *Pseudomonas aeruginosa* has intrinsic antibiotic resistance mechanisms and determinants for disinfectant resistance (Murray et al., 2015). Due to its natural high adaptability, *P. aeruginosa* is one of the most common pathogens in hospitals (de Abreu et al., 2014; Reynolds and Kollef, 2021). The WHO and CDC list multidrug-resistant *P. aeruginosa* as one of the greatest threats to the global health care system and have called for the urgent need to develop new antibiotics against this pathogen (WHO, 2017; CDC, 2019). In 2017, multidrug-resistant *P. aeruginosa* caused more than 30,000 infections in the US with a mortality rate of 10%, producing estimated economic losses of $800 million (CDC, 2019). Moreover, *P. aeruginosa* is particularly relevant as the second most common cause of catheter-associated urinary tract infections (CAUTIs) (Sievert et al., 2013; Weiner et al., 2016; Weiner-Lastinger et al., 2020), causing more than 14,000 cases, 14% of all CAUTIs between 2015 to 2017 (CDC, 2019).

**Figure 1:**
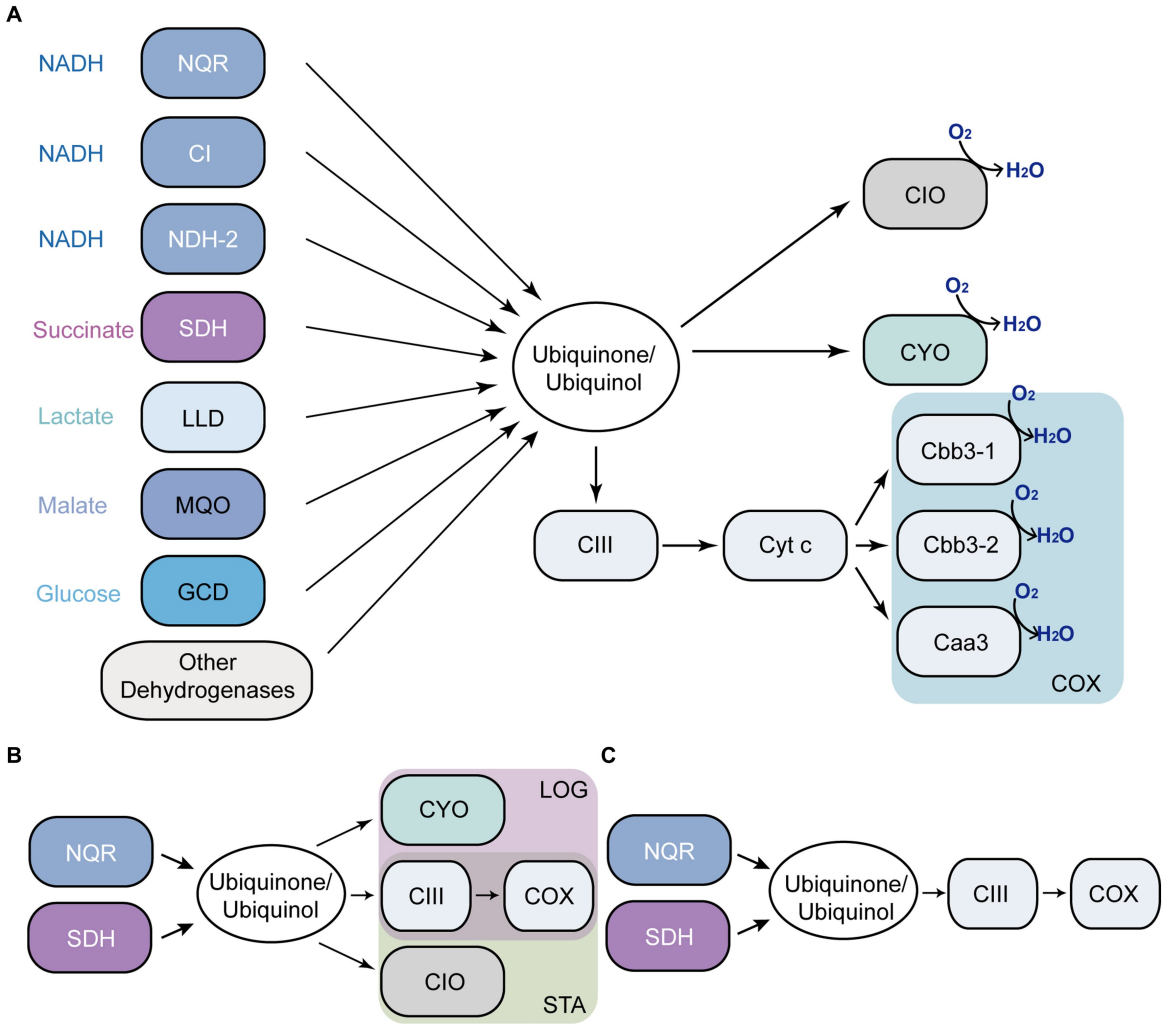
*Pseudomonas aeruginosa* aerobic respiratory chain. Respiratory enzymes annotated in *P. aeruginosa* genome **(A)**. Main components of the respiratory chain in LB **(B)**. mAUM **(C)**. NQR, ion-pumping NADH: ubiquinone oxidoreductase; CI, complex I; NDH-2, NADH: ubiquinone oxidoreductase II; SDH, succinate dehydrogenase; LLD, L-lactate dehydrogenase; MQO, malate: quinone oxidoreductase; GCD, glucose dehydrogenase; CIII, complex III (the bc1 complex); CIO, cyanide-insensitive *bd* oxidase; CYO, *bo*_3_-type oxidase; COX, three cytochrome c oxidases; LOG, logarithmic phase; STA, stationary phase.

*Pseudomonas aeruginosa* has an unparalleled ability to adapt to different environmental conditions, due to its large genome, coding for a variety of virulence factors and for a greatly adaptable metabolism, and regulation capacity (Stover et al., 2000; Mikkelsen et al., 2011). *Pseudomonas aeruginosa* contains one of the most complex and highly branched respiratory chains (Figure 1A) that includes 17 predicted dehydrogenases, five aerobic and two anaerobic terminal oxidases, providing the flexibility for ATP generation that allows its survival under a variety of environments (Williams et al., 2006). *Pseudomonas aeruginosa* contains three seemingly redundant NADH dehydrogenases that catalyze electron transfer form NADH to ubiquinone: Complex I, NDH-2 and NQR (Torres et al., 2019; Liang et al., 2020). Complex I couples NADH oxidation to proton pumping and contains 14–15 subunits (nuoA-N) (Yagi, 1991; Baradaran et al., 2013). The non-proton pumping NDH-2 is coded by a single gene and contains FAD as its sole cofactor (Yagi, 1991; Kerscher et al., 2008). NQR is encoded by nqrA-F genes and contains six confirmed cofactors (Juárez and Barquera, 2012; Reyes-Prieto et al., 2014; Raba et al., 2018), and in this microorganism it functions as a proton pump (Raba et al., 2018), rather than a sodium pump as in other cases (Reyes-Prieto et al., 2014; Dibrov et al., 2017b). Succinate dehydrogenase (SDH), lactate dehydrogenase, malate dehydrogenase, glucose dehydrogenase and other dehydrogenases provide electrons to the respiratory chain to adapt to different environmental conditions (Kretzschmar et al., 2002; Frimmersdorf et al., 2010; Wang et al., 2018; McGill et al., 2021). In addition to the diversity of dehydrogenases, *P. aeruginosa* contains five aerobic terminal oxidases, including three COX-type oxidases: one aa3 -type oxidase (homologous to mitochondrial complex IV) and two cbb3 -type oxidases (Jo et al., 2014), which are connected to the ubiquinol pool through complex III. *Pseudomonas aeruginosa* also contains two ubiquinol oxidases: a bo3-type oxidase (CYO), which contains 5 subunits (Arai, 2011), and a cyanide-insensitive bd oxidase (CIO), containing two subunits (Arai et al., 2014; Jo et al., 2014).

The main aim of the study is to understand the metabolic adaptations of *P. aeruginosa* to the urine. In a previous report, we characterized the function of *P. aeruginosa* respiratory enzymes in the logarithmic phase of growth of cells cultured in modified artificial urine media (mAUM), which was recently developed by our group and offers a reliable and stable medium to study the bacterial adaptations to urine (Liang et al., 2020). In this report, we have expanded the previous studies to the stationary phase of growth, as this condition might represent a relevant stage in CAUTIs and UTIs in general, since the majority of biofilm colonizers are static organisms (Paula et al., 2020). Our results offer a clear picture of the role and relevance of the metabolic pathways and respiratory enzymes employed by this microorganism to survive in urine-like media (Figure 1C). Moreover, the data reveal that NQR, SDH and complex III play critical roles in cell physiology and represent potential antibiotic targets against CAUTIs produced by *P. aeruginosa* (Figures 1B,C).

## 2 Materials and methods

### 2.1 Bacterial growth and membrane purification

*Pseudomonas aeruginosa* PAO1 was grown in LB media and in freshly-prepared mAUM (Table 1; Supplementary Figure S1; Liang et al., 2020) in 2 L flasks. Cultures were initiated by diluting overnight cultures (1:1000) in 1 L of media. Cells were grown at 37°C in baffled flasks and agitated at 250 rpm. Cultures were collected at the logarithmic (7 h) and stationary phases of growth (24 h in LB broth and 12 h in mAUM). Cells were harvested and washed twice in KHE buffer (150 mM KCl, 20 mM HEPES, 1 mM EDTA, pH 7.5). Bacteria cells were disrupted at 16,000 psi using the Avestin Emulsiflex-C5. The cell suspension was centrifuged at 10,000 × *g* to eliminate cell debris. The membrane fraction was obtained by ultracentrifugation at 100,000 × *g*. The membrane pellet was washed with KHE buffer and stored at −80°C.

**Table 1:** Composition of modified artificial urine media (mAUM) (pH 6.5).

Component	Concentration
EDTA	3 mM
Citric acid	2 mM
CaCl_2_	2.5 mM
NaCl	80 mM
Na_2_SO_4_	10 mM
MgCl_2_	5 mM
KH_2_PO_4_	50 mM
Uric acid	0.23 mM
NaHCO_3_	37 mM
Bovine peptone	1 (g/l)
Yeast extract	0.5 (g/L)
Urea	170 mM
Creatinine	7 mM
NH_4_Cl	25 mM
Sodium lactate	1 mM
FeSO_4_	3.5 μM

Wild type PAO1 was obtained from Dr. John C. Alverdy, University of Chicago. Growth of wild-type *P. aeruginosa* was compared to that of the Δbc1, ΔcioB, ΔccoN-1 and ΔccoN-2 mutants. The mutants were obtained from the *P. aeruginosa* PA14 Transposon Insertion Mutants Library (Liberati et al., 2006), kindly provided by Dr. Martin Schuster and Dr. Claudia Hässe from Oregon State University. Cultures of PAO1 wild type strains and the mutants of PA14 were grown in LB and mAUM at an inoculation ratio of 1:40 in 4 mL and incubated at 37°C under vigorous agitation, 250 rpm. Growth curves were analyzed using a logistic function to calculate the yield of the growth, the growth rate and the time of lag phase (Kahm et al., 2010).

### 2.2 Respiratory activity

Respiratory activities were measured in *P. aeruginosa* membranes (0.2 mg/mL) using a Clark-type oxygen electrode (YSI 5300) in a customized oximetric chamber (Liang et al., 2018, 2020). Experiments were carried out in KHE buffer at 37°C with different substrates, including 200 μM NADH, 20 mM succinate, 10 mM L-malate, 10 mM lactate (racemic mixture) and 10 mM D-glucose. Ubiquinol oxidase was measured in the presence of 50 μM ubiquinone-1 and 500 μM DTT (1,4-Dithiothreitol), which rapidly reduces ubiquinone (Chu et al., 2006; Furt et al., 2010; Gong et al., 2018). Cytochrome c oxidase activity was measured using 1 mM ascorbate and 100 μM TMPD (N,N,N′,N′-tetramethyl-para-phenylene-diamine) (Kimelberg and Nicholls, 1969). Cyanide titrations (0.1 μM to 10 mM) of the terminal oxidases were measured using NADH as a substrate with freshly prepared KCN.

### 2.3 NADH dehydrogenase activity

NADH: ubiquinone oxidoreductase activity (Q_red_) was determined in membranes in the presence of NADH or deamino-NADH with or without 1 μM rotenone to inhibit complex I, in KHE buffer containing 0.05% DDM (β-D-dodecyl maltoside), 50 mM ubiquinone-1 and 10 mM KCN to inhibit terminal oxidases (Matsushita et al., 1987; Managò et al., 2015; Liang et al., 2020). The activity was followed spectrophotometrically at 282 nm as described previously (Juárez et al., 2011), using an Agilent Cary 8,454 UV–vis spectrophotometer. The activities reported here represent initial rates, collected within two minutes after the reaction was initiated. The Q_red_ % was normalized comparing the individual dehydrogenase activities to the total activity by all dehydrogenases.

### 2.4 Blue native PAGE

Blue native polyacrylamide gel electrophoresis (BN-PAGE) was used to separate native and functional *P. aeruginosa* respiratory enzymes (complex I, NDH-2 and NQR) based on their molecular weights (Wittig et al., 2006). *Pseudomonas aeruginosa* membrane samples were washed and resuspended in buffer containing 50 mM Bis-Tris, 500 mM aminocaproic acid, pH 7.0, and were solubilized at 4°C for 40 min, using b-D-dodecylmaltoside at a detergent: protein ratio of 2, as described previously (Liang et al., 2020). Solubilized membranes (50 μg of protein) were loaded into a 4 to 16% gradient acrylamide-bisacrylamide gel. NADH dehydrogenase activity was assayed in-gel with MTT as electron acceptor using NADH as the substrate as described previously (Liang et al., 2020). The reaction buffer contained 100 mM Tris, 140 μM NADH, 50 μM MTT (3-(4,5-Dimethylthiazol-2-yl)-2,5-Diphenyltetrazolium Bromide), pH 7.4 (Liang et al., 2020). The Coomassie-blue was then washed off the gel by 0.1% SDS after the MTT staining. The reduction of MTT by the NADH dehydrogenase produces a purple color on the location of the enzyme bands.

### 2.5 RNA isolation and cDNA synthesis

Bacteria were collected at the stationary phase in LB and mAUM, and were suspended in Purelink RNA Mini Kit lysis buffer, containing 10 mg/mL lysozyme, 10% SDS and 1% 2-mercaptoethanol. The lysate was homogenized by passing it through a 21-gauge needle 5 times. The supernatant was collected after centrifugation at 12,000 × *g* for 2 min at room temperature. Total RNA was extracted using the Purelink RNA Mini Kit, according to the manufacturer’s instructions (ThermoFisher Scientific, Waltham, MA). RNA concentration was measured by AccuBlue Broad Range RNA Quantitation Kit (Biotium, Fremon, CA) on a Qubit 3.0 fluorometer. cDNA was synthesized with The ProtoScript II First Strand cDNA Synthesis Kit (New England Biolabs Ipswich, MA), using the random primer mix.

### 2.6 Real time quantitative PCR

RT-qPCR was performed following the guidelines for minimum information of quantitative real-time PCR for publication (Bustin et al., 2009). The rpoS gene was selected as reference, which shows transcriptional stability under carbon starvation (Alqarni et al., 2016). The forward and reverse primers (Table 2) were designed using the Primer3 software (Kõressaar et al., 2018). 100 ng cDNA were used as template in each amplification. Amplification and quantification were done by the fluorescent dye-based double-strand DNA detective PCR technology, using the iTaq Universal SYBR Green Supermix kit (Bio-Rad, Hercules, CA). The real-time PCR reaction was run in a Bio-Rad CFX Connect Real-Time PCR Detection System following the manufacturer’s instructions (Bio-Rad). The relative quantification of PCR expression was analyzed by the 2-ΔΔCT method that compared the differences between the cycle threshold of target gene and reference gene (rpoS) under the control condition (LB) and experimental condition (mAUM) (Livak and Schmittgen, 2001).

**Table 2:** RT-qPCR primers used in this study.

Target gene	Gene No.	Sequence 5′ – 3’	Amplicon size (bp)	Gene description	Sources
*nuoG*	PA2642	TGGCCACTATCCACGTAGAC TTCTCGTCGGTGTACTGCTT	169	NADH-quinone oxidoreductase subunit G	This study
*nqrA*	PA2999	GATAAAACGTGGCCTGGACC GCTGACGGAGGGATTCTTCT	193	NADH-quinone reductase subunit A	This study
*ndh*	PA4538	CTGCTGGTGAAATCGCTACG GATACGTAGAACATCCGCGC	176	NADH dehydrogenase	This study
*rpoS*	PA3622	CTCCCCGGGCAACTCCAAAAG CGATCATCCGCTTCCGACCAG	197	RNA polymerase sigma factor, housekeeping	Alqarni et al. (2016)

### 2.7 Proteomics

Bacterial membrane samples (20 μg of protein) were prepared using the Filter Aided Sample Preparation protocol, employing 30 K NMWL centrifugal filter units. Samples were reduced with 20 mM dithiothreitol, followed by 20 min alkylation with 50 mM iodoacetamide. The alkylated proteins underwent three washes with 200 μL of 8 M Urea in 0.1 M Ammonium Bicarbonate (ABC) and were subsequently equilibrated three times with 0.1 M ABC prior to trypsin digestion. Proteins were digested using Trypsin at an enzyme: protein ratio of 1:50 in 50 μL of 0.1 M ABC buffer at 37°C overnight. The resulting digested peptides were collected by centrifugation at 14,000 *g* for 20 min, followed by two elutions with 50 μL of 50 mM ABC and one elution with 50 μL of 0.5 M NaCl. The eluted peptides were desalted using C18 columns and were dried. The digested proteins were then reconstituted in a 30 μL solution of 5% acetonitrile and 0.1% formic acid buffer for LC–MS analysis.

Five microliters of digested peptides were analyzed using a Q Exactive HF mass spectrometer coupled to an UltiMate 3,000 RSLC nanosystem, featuring a Nanospray Flex Ion Source (Thermo Fisher Scientific). The digested peptides were initially loaded into a Waters nanoEase M/Z C18 (100 Å, 5 μm, 180 μm × 20 m) trap column and subsequently separated using a 75 μm × 150 mm Waters BEH C18 (130 Å, 1.7 μm, 75 μm × 15 cm) column, at a flow rate of 300 nL/min. Solvent A consisted of 0.1% formic acid (FA) in water, while solvent B comprised 0.1% FA and 80% acetonitrile in water. The liquid chromatography gradient profile started with 5% of solvent B from 0 to 3 min, increased to 8% B at 3.2 min, ramped from 8 to 35% B within 110 min, elevated from 35 to 95% B in 115 min, underwent a 95% wash until 119.8 min, and finally equilibrated back to 5% B until 130 min.

Full MS scans were acquired using the Q Exactive mass spectrometer over the range of 350 to 1,400 m/z, operating at a resolution of 60,000 (at 200 m/z) from 10 min to 130 min. The AGC (automatic gain control) target value was set at 3×10^6^ for the full scan. The top 15 most intense peaks with charge states 2, 3, 4, and 5 were subjected to fragmentation in the HCD (higher-energy collisional dissociation) collision cell with a normalized collision energy of 28%. These peaks were subsequently excluded for the next 30 s within a mass window of 1.4 m/z. Tandem mass spectra were collected in the mass analyzer at a resolution of 30,000, with an AGC target value set at 1×10^5^. The ion selection threshold was established at 1×10^4^ counts, while the maximum allowed ion injection time was 50 msec for both full scans and fragment ion scans. Mass spectrometry data were deposited in the MassIVE database under the accession number PXD047660.

Spectra data were searched against the Uniprot *P. aeruginosa* database using Mascot daemon (2.6.0, updated on 08/11/20). Results were entered into Scaffold Q + S software (v5.1.2, Proteome Software, Portland, OR) for compilation, normalization, and comparison of spectral counts, etc. The filtering criteria of protein identification were a 1% false discovery rate (FDR, statistical measure of the certainty of protein identification) of protein and peptide with 1 minimum peptide count. Peptide sequences and their assigned UniProt accessions were parsed from the peptide report with parse_scaffold_peptide_summary.py v0.1.0. Protein sequences and enzyme classification (EC) numbers for each UniProt accession were downloaded utilizing the Consortium’s API as implemented in grab_UniProt_data.py v0.3.0. The KEGG (Kanehisa et al., 2023) organism code for *P. aeruginosa* PAO1 (pae) was obtained from the KEGG organism catalog.^1^ KEGG pathways were reconstructed from mass spec data using the MassSpec2KEGG pipeline^2^ as described below. UniProt accessions were converted to KEGG orthologs (KOs) leveraging the KEGG API as implemented in UniProt_to_KEGG.py v0.5.0 as follows. UniProt accessions were mapped to NCBI accessions by performing DIAMOND (Buchfink et al., 2021) BLASTP homology searches against the *P. aeruginosa* PAO1 reference proteome (NCBI accession number GCF_000006765.1). NCBI accessions were converted to *P. aeruginosa* PAO1 specific KEGG IDs with the *P. aeruginosa* PAO1 specific KEGG ID/NCBI Protein ID conversion file from the KEGG database.

## 3 Results

### 3.1 Proteomic analysis

**Figure 2:**
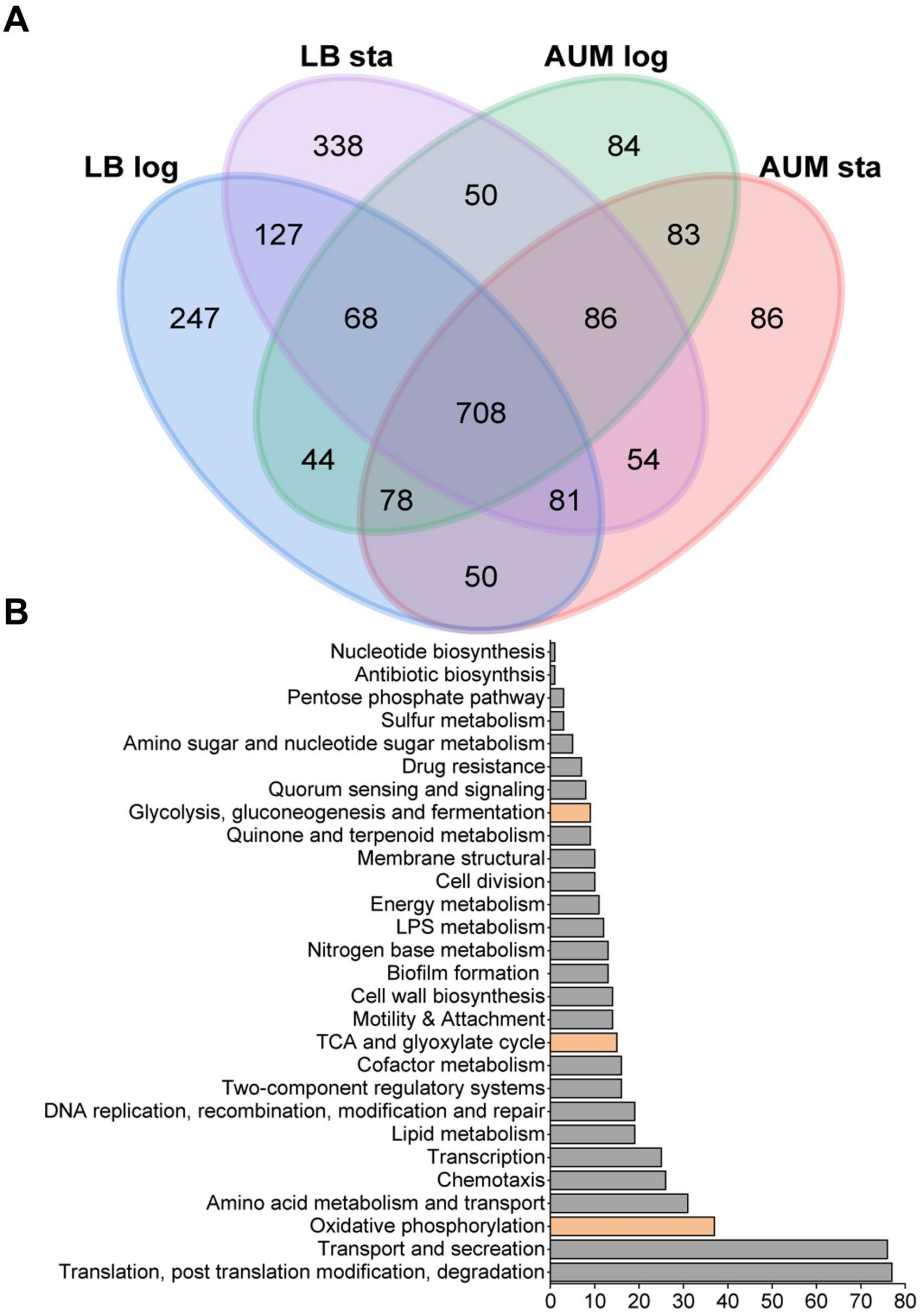
Proteomic data from *P. aeruginosa* PAO1 membrane isolated samples grown in LB and mAUM media. **(A)** Venn diagram of the four conditions depicting the numbers and overlaps of differentially identified proteins. **(B)** Bar graph showing the distribution of the protein function among the 708 core proteins, including those of the central carbon metabolism, indicated in orange. Hypothetical proteins are not included.

To characterize the metabolic strategies used by *P. aeruginosa* when grown in LB or mAUM, we carried out an analysis of the membrane and membrane-associated proteins found in the logarithmic and the stationary phases in these media. Of the 5,570 possible ORFs, a total of 2,184 proteins were identified in the four conditions, employing a stringent filtering threshold of 1% FDR for both peptide and protein recognition. In mAUM, 1,201 proteins were identified in mid-log growth phase and 1,226 proteins were identified in the stationary phase. In LB medium, 1,403 proteins were detected in the mid-log phase and 1,512 proteins in the stationary phase (Supplementary Data 1). Protein expression patterns are shown in a Venn diagram (Figure 2A), which illustrates the proteins identified across these conditions. Our data reveals 708 membrane or membrane-associated proteins that are recognized as core proteins, common among all growth conditions. Figure 2B illustrates the role of the core proteins, evaluated from the analysis of KEGG pathways, GO terms, and the Pseudomonas Community Annotation project (Winsor et al., 2016). As can be observed in Table 3, aerobic oxidative phosphorylation, the Entner-Duodoroff pathway, the pentose phosphate pathway, and the Krebs cycle/ glyoxylate shunt are core pathways in *P. aeruginosa*.

**Table 3:** Metabolic proteins related to Entner-Duodoroff pathway, gluconeogenesis, fermentation, pentose phosphate pathway, Krebs cycle, and glyoxylate cycle.

	Locus tag	RefSeq accession	Gene length	Gene name	Enzyme	Protein name
Entner Duodoroff, gluconeogenesis, and fermentation	PA4732	NP_253420	1,665	*pgi*	Glucose-6-P-isomerase	Glucose-6-phosphate isomerase
	PA3001	NP_251691	1,386		Glyceraldehyde-3-P-dehydrogenase	Glyceraldehyde-3-phosphate dehydrogenase
	PA1770	NP_250461	2,376	*ppsA*	Phosphoenolpyruvate synthase	Phosphoenolpyruvate synthase
	PA3471	NP_252161	1,695		Malic enzyme	NAD-dependent malic enzyme
	PA5046	NP_253733	1,269			Malic enzyme
	PA4771	NP_253459	1,146	*lldD*	Lactate dehydrogenase	L-lactate dehydrogenase
	PA4812	YP_003933614	3,081	*fdnG*		Formate dehydrogenase-O major subunit
	PA5322	NP_254009	1,392	*algC*		Phosphomannomutase
Krebs cycle and glyoxylate cycle	PA5015	NP_253702	2,649	*aceE*	Pyruvate dehydrogenase	Pyruvate dehydrogenase subunit E1
	PA5016	NP_253703	1,644	*aceF*		Dihydrolipoamide acetyltransferase
	PA1587	NP_250278	1,437	*lpd*		2-oxoglutarate dehydrogenase complex dihydrolipoyl dehydrogenase
	PA1787	NP_250478	2,610	*acnB*	Aconitate synthase	Aconitate hydratase B
	PA2623	NP_251313	1,257	*icd*	Isocitrate dehydrogenase	Isocitrate dehydrogenase
	PA2624	NP_251314	2,226	*idh*		Isocitrate dehydrogenase
	PA1585	NP_250276	2,832	*sucA*	2-Ketoglutarate dehydrogenase	2-oxoglutarate dehydrogenase subunit E1
	PA1586	NP_250277	1,230	*sucB*		2-oxoglutarate dehydrogenase complex dihydrolipoyllysine-residue succinyltransferase
	PA1588	NP_250279	1,167	*sucC*	Succinyl-coenzyme A synthetase	Succinyl-CoA ligase subunit beta
	PA1589	NP_250280	888	*sucD*		Succinyl-CoA ligase subunit alpha
	PA1583	NP_250274	1773	*sdhA*	Succinate dehydrogenase	Succinate dehydrogenase flavoprotein subunit
	PA1584	NP_250275	708	*sdhB*		Succinate dehydrogenase iron–sulfur subunit
	PA1582	NP_250273	369	*sdhD*		Succinate dehydrogenase subunit D
	PA3452	NP_252142	1,572	*mqoA*	Malate:quinone oxidoreductase	Malate:quinone oxidoreductase
	PA4640	NP_253330	1,524	*mqoB*		Malate:quinone oxidoreductase

The respiratory proteins identified across all growth conditions are detailed in Table 4, comprising a total of 12 enzymes. Our data shows that *P. aeruginosa* expresses the three NADH dehydrogenases: NQR, complex I, and NDH-2, in all conditions. SDH, complex III, CYO oxidase, along with the two cbb3 oxidases are also core proteins. Additionally, lactate dehydrogenase (lld, PA4771) and malate dehydrogenase (mqo) are consistently found across all growth conditions, as well as F_1_-F_0_ ATP synthase. Interestingly, the A-type cytochrome c oxidase (coxB, PA0105) was identified during mAUM growth in the stationary phase (Supplementary Data 2). This enzyme is considered more energy efficient compared to other oxidases (Osamura et al., 2017). Its participation in these conditions could be beneficial considering the limited nutrients in the media. The cyanide-insensitive CIO oxidase peptides (cioA, PA3930) were identified in LB stationary phase as well as in AUM growth (Supplementary Data 2), aligning with increased cyanide tolerance in *P. aeruginosa* cultivated in AUM. Correspondingly, the early stages of LB growth provide enough nutrients for *P. aeruginosa* which will not trigger the production of cyanide and the expression of CIO. An intriguing discovery is the presence of glucose dehydrogenase (gcd, PA2290, Supplementary Data 2) across all four growth conditions, despite the absence of glucose in mAUM media. This suggests an activated gluconeogenesis pathway that potentially generates glucose as an energy source. Overall, the proteomics data show that *P. aeruginosa* carries a very active aerobic respiratory metabolism in urine-like media, which was further characterized in this report.

**Table 4:** Respiratory proteins identified among all growth conditions tested for *Pseudomonas aeruginosa* PAO1.

Locus tag	RefSeq accession	Gene length	Gene name	Enzyme	Protein name
PA2637	NP_251327	414	*nuoA*	Complex I	NADH-quinone oxidoreductase subunit A
PA2638	NP_251328	678	*nuoB*		NADH-quinone oxidoreductase subunit B
PA2639	NP_251329	1782	*nuoD*		NADH:-quinone oxidoreductase subunit C/D
PA2640	NP_251330	501	*nuoE*		NADH-quinone oxidoreductase subunit E
PA2641	NP_251331	1,347	*nuoF*		NADH dehydrogenase I subunit F
PA2642	NP_251332	2,718	*nuoG*		NADH-quinone oxidoreductase subunit G
PA2643	NP_251333	996	*nuoH*		NADH-quinone oxidoreductase subunit H
PA2644	NP_251334	549	*nuoI*		NADH-quinone oxidoreductase subunit I
PA2647	NP_251337	1848	*nuoL*		NADH-quinone oxidoreductase subunit L
PA2649	NP_251339	1,461	*nuoN*		NADH-quinone oxidoreductase subunit N
PA2999	NP_251689	1,338	*nqrA*	nqr	Na(+)-translocating NADH-quinone reductase subunit A
PA2997	NP_251687	786	*nqrC*		Na(+)-translocating NADH-quinone reductase subunit C
PA2994	NP_251684	1,224	*nqrF*		Na(+)-translocating NADH-quinone reductase subunit F
PA4538	NP_253228	1,308	*ndh*	ndh-2	NADH dehydrogenase
PA1583	NP_250274	1773	*sdhA*	sdh	Succinate dehydrogenase flavoprotein subunit
PA1584	NP_250275	708	*sdhB*		Succinate dehydrogenase iron–sulfur subunit
PA1582	NP_250273	369	*sdhD*		Succinate dehydrogenase subunit D
PA4429	NP_253119	783		bc1 complex	Cytochrome C1
PA4430	NP_253120	1,212			Cytochrome b
PA4431	NP_253121	594			Iron–sulfur protein
PA1552	NP_250243	957	*ccoP1*	cbb3-1	Cytochrome C oxidase cbb3-type subunit CcoP
PA1553	NP_250244	612	*ccoO1*		cbb3-type cytochrome C oxidase subunit II
PA1554	NP_250245	1,428	*ccoN1*		cbb3-type cytochrome C oxidase subunit I
PA1556	NP_250247	609	*ccoO2*	cbb3-2	cbb3-type cytochrome C oxidase subunit II
PA1317	NP_250008	996	*cyoA*	cyoA	Cytochrome o ubiquinol oxidase subunit II
PA2290	NP_250980	2,412	*gcd*	gcd	Glucose dehydrogenase
PA4771	NP_253459	1,146	*lldD*	lldD	L-lactate dehydrogenase
PA4640	NP_253330	1,524	*mqoB*	mqo	Malate:quinone oxidoreductase
PA3452	NP_252142	1,572	*mqoA*		Malate:quinone oxidoreductase
PA0139	NP_248829	564	*ahpC*		Alkyl hydroperoxide reductase
PA1475	NP_250166	702	*ccmA*		Cytochrome c biogenesis ATP-binding export protein CcmA
PA2991	NP_251681	1,395	*sth*		Soluble pyridine nucleotide transhydrogenase
PA3446	NP_252136	594			NAD(P)H-dependent FMN reductase
PA3768	NP_252457	1,392			Metallo-oxidoreductase
PA5242	NP_253929	2,211	*ppk*		Polyphosphate kinase
PA5553	NP_254240	426	*atpC*	ATP synthase	ATP synthase subunit epsilon
PA5554	NP_254241	1,377	*atpD*		ATP synthase subunit beta
PA5555	NP_254242	861	*atpG*		ATP synthase subunit gamma
PA5556	NP_254243	1,545	*atpA*		ATP synthase subunit alpha
PA5557	NP_254244	537	*atpH*		ATP synthase subunit delta
PA5558	NP_254245	471	*atpF*		ATP synthase subunit B
PA5560	NP_254247	870	*atpB*		ATP synthase subunit A

### 3.2 Dehydrogenase and oxidase activities

**Figure 3:**
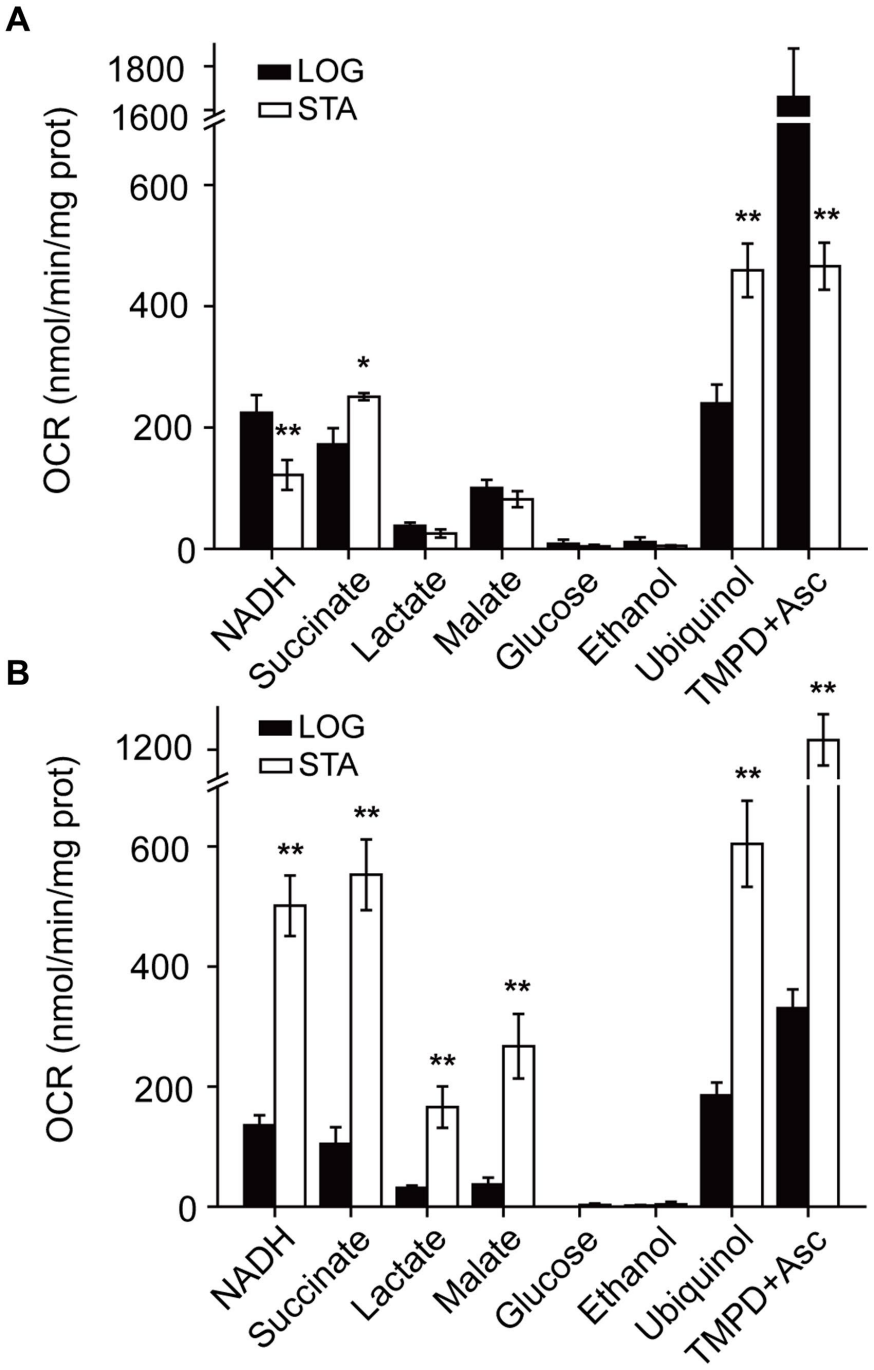
*Pseudomonas aeruginosa* respiratory activity in LB and mAUM membranes. Oxygen consumption rate (OCR) was measured in bacterial membranes grown in LB **(A)** and mAUM **(B)** collected in logarithmic (black bars) and stationary (white bars) phases. The data from the logarithmic phase was obtained from a previous publication (Liang et al., 2020). Respiratory activity was measured in KHE buffer with the following substrates: 200 μM NADH, 10 mM succinate, 10 mM lactate, 10 mM glucose, 0.3% ethanol, 50 μM ubiquinol or 100 μM TMPD+ plus 5 mM ascorbic acid. *n* > 3 independent experiments for each group. Data represented as mean ± SD. *p < 0.05 and **p < 0.01 determined by *t*-test of the logarithmic versus stationary phase.

In a previous report, we characterized the respiratory chain of *P. aeruginosa* in the logarithmic phase of growth in conditions resembling urine, in mAUM media (Liang et al., 2020). However, in CAUTIs the growth rate of bacteria is likely low (Chambers et al., 1999), and the physiology of the cells might be similar to the stationary phase of growth in this and other bacteria (Trautner and Darouiche, 2004; Jacobsen et al., 2008). Thus, we aimed to characterize the *P. aeruginosa* respiratory chain in the stationary phase. *Pseudomonas aeruginosa* PAO1 plasma membranes of cells grown in mAUM were obtained in the stationary phase, where most of the cells are no longer dividing or dividing at a reduced rate and before the death phase has started. The respiratory activity was measured as the oxygen consumption rate in the presence of different substrates such as NADH, succinate, lactate, malate, glucose, and ethanol, to evaluate different dehydrogenases. In LB media, the activity of NADH dehydrogenases decreased by half in the stationary stage compared to the rapid growing stage (Figure 3A). On the other hand, in mAUM the activity of the NADH dehydrogenases increased more than threefold in the stationary stage compared to the rapid growth phase (Figure 3B). Succinate oxidation also increased in LB and mAUM in the stationary phase, with the highest increase in mAUM (>five-fold). Lactate and malate oxidation were not significantly modified in LB but increased four times in mAUM in the stationary phase. As reported before, glucose and ethanol oxidation did not contribute substantially to the respiratory activity (Liang et al., 2020).

In addition to the dehydrogenases, the activity of the terminal aerobic oxidases was investigated using ubiquinol and TMPD-ascorbate as substrates. Ubiquinol oxidation reports the activity of CIO and CYO oxidases, as well as the activity of the pathway composed by complex III and COX oxidases, such as cytochrome aa3 and cbb3 oxidases (Liang et al., 2020), and TMPD-ascorbate oxidation reports the activity of cytochrome C oxidases directly (Liang et al., 2020). In cells in the stationary phase grown in LB media, the activity of aerobic ubiquinol oxidases doubled, while the activity of cytochrome C oxidases decreased by >3-fold (Figure 3A). In the stationary phase, the cells grown in mAUM show a 3-fold activation of the oxidases (Figure 3B). Overall, in urine-like media, *P. aeruginosa* undergoes deep metabolic changes in the stationary phase, which include a 3–4 -fold activation of the respiratory activity.

### 3.3 Participation of NADH dehydrogenases in respiratory activity

**Figure 4:**
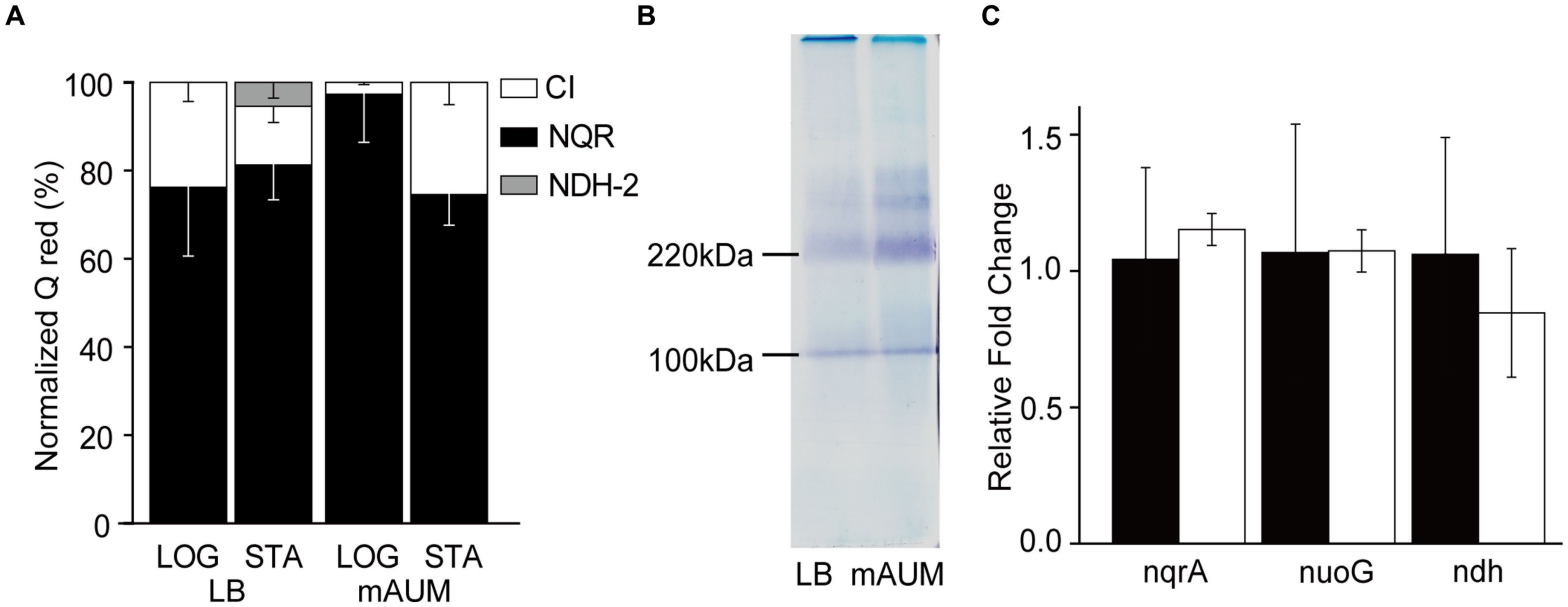
Participation of NADH dehydrogenases in *P. aeruginosa* metabolism in LB and mAUM media. **(A)** Normalized activity of the three NADH dehydrogenases in logarithmic (LOG) and stationary (STA) phases of cells grown in LB and mAUM media. The data from the logarithmic phase was obtained from a previous publication (Liang et al., 2020). **(B)** In-gel activity staining of NADH dehydrogenases in BN-PAGE. **(C)** Relative expression of the three NADH dehydrogenases, as determined by quantitative PCR in stationary phase of cells grown in LB (black bars) and mAUM (white bars).

The participation of NADH dehydrogenases in the respiratory activity of stationary phase *P. aeruginosa* cells was also characterized. Although the three NADH dehydrogenases coded in the genome catalyze the same chemical reaction: electron transfer from NADH to ubiquinone, they can be differentiated by their substrate selectivity and inhibitor sensitivity. Complex I is able to use as substrates NADH and deamino-NADH equally well (Matsushita et al., 1987), and it is specifically inhibited by rotenone (Degli Esposti, 1998; Zickermann et al., 2000). NQR also uses NADH and deamino-NADH, but it is insensitive to rotenone (Zhou et al., 1999). On the other hand, NDH-2 exclusively uses NADH as substrate and is insensitive to rotenone (Matsushita et al., 1987; Zambrano and Kolter, 1993). Our results show that in all conditions tested, the NADH and deamino-NADH oxidation were very similar and were largely insensitive to rotenone, indicating that NQR is the main functional NADH dehydrogenase, sustaining 75–95% of the electron transfer, with a modest participation of complex I and a negligible function of NDH-2 (Figure 4A).

### 3.4 Blue native gel electrophoresis

In addition to the substrate specificity, the three NADH dehydrogenases can be differentiated by their sizes. Complex I, NDH-2 and NQR have an estimated molecular weight of 530, 46, and 210 kDa, respectively (Liang et al., 2020), and can be separated electrophoretically using Blue Native PAGE, as described previously (Liang et al., 2020). The complexes can be stained in-gel following the precipitation of the electron acceptor MTT using NADH as substrate (Berridge et al., 2005). As shown in Figure 4B, the gel contains a prominent band of 210 kDa, which corresponds to NQR, as confirmed previously by our group using mass spectroscopy (Liang et al., 2020). This band increases 3.3-fold in the mAUM stationary phase membranes, consistent with the increase in respiratory activity discussed above. Another band of 110 kDa is also visible but as described before (data not shown), it does not contain any NADH dehydrogenase peptides (Liang et al., 2020), and does not show an increased intensity in the log state cells. Bands of 370 and 300 kDa were also evident in the gel, which may correspond to complex I subcomplexes. However, they have low intensity and required much longer to be stained, consistent with the low activity or complex I in these samples.

### 3.5 NADH dehydrogenase mRNA expression

To understand the genetic regulation of the respiratory chain of *P. aeruginosa* grown in LB and mAUM, we carried out a quantification of mRNAs of the three NADH dehydrogenases through qPCR, using the *rpoS* gene as a reference gene, which presents stability under carbon-limiting conditions (Alqarni et al., 2016) to normalize the results. Figure 4A shows that although there are changes in the activities of the three dehydrogenases depending on the media and the growth stage, there are no significant differences in the expression of these genes at the mRNA level (Figure 4C), indicating that other levels of genetic regulation may play major roles in the control of the respiratory activity *in vivo*.

### 3.6 Contribution of terminal oxidases to respiratory activity

**Figure 5:**
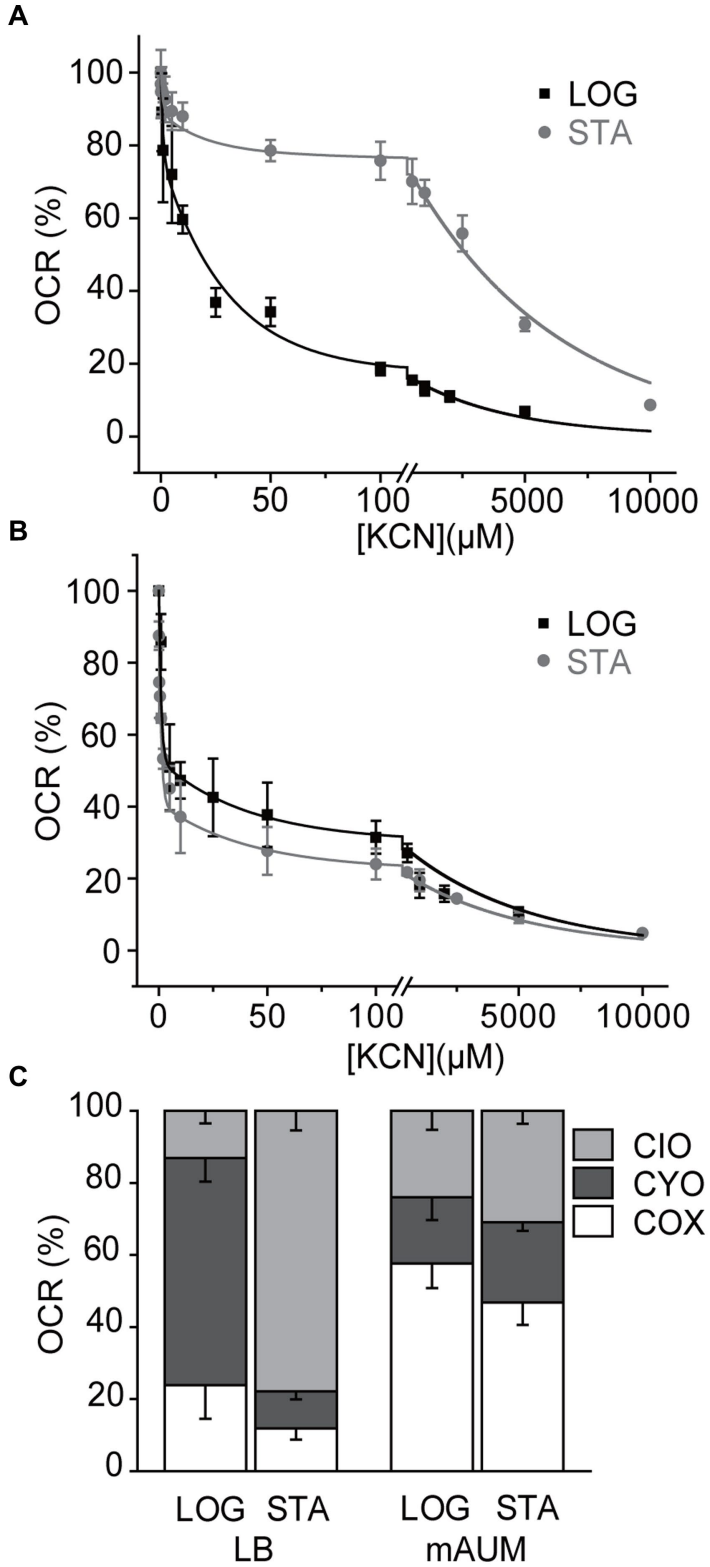
Participation of terminal oxidases in *P. aeruginosa* metabolism in LB and mAUM media. KCN titration of the NADH-dependent respiratory activity of LB **(A)** and mAUM **(B)** membranes collected from logarithmic (black) and stationary (gray) phase cells. The data from the logarithmic phase was obtained from a previous publication (Liang et al., 2020). **(C)** Relative contribution of the oxidases to the respiratory activity of cells grown in LB (left) and mAUM (right) under logarithmic and stationary phases.

As mentioned above, *P. aeruginosa* contains five different aerobic oxidases: CIO oxidase, CYO oxidase, and the pathway composed of complex III and cytochrome oxidases aa3, cbb3-1 and cbb3-2. Although these enzymes catalyze the same reaction: electron transfer from ubiquinol to oxygen, they can be differentiated by their sensitivity to cyanide. In a previous study we have shown that by conducting a KCN titration, the contribution of each oxidase can be unambiguously determined, since the IC_50_ for cytochrome oxidases (<1 μM), CYO oxidase (10–30 μM), and CIO oxidase (>5 mM) for cyanide are vastly different (Liang et al., 2020). KCN titrations were carried out in membranes obtained in the stationary phase of growth and the data was fitted to a three component Michaelis–Menten inhibition equation (Figures 5A,B) to obtain the contributions of each oxidase, as reported previously (Liang et al., 2020). In LB media we have reported that the main oxidase in the logarithmic phase is CYO oxidase. However, when the cells enter the stationary phase, the respiratory chain changes dramatically, with close to 80% of the activity carried by the cyanide-insensitive CIO oxidase (Figure 5C). Even though a three-fold increase in the respiratory activity was induced in the stationary phase of growth in cells grown in mAUM, the contributions of the different oxidases did not change significantly, with complex III and the COX oxidases contributing to 50% of the respiratory activity, compared to close to 60% in the log phase (Figure 5C).

### 3.7 Studies on mutants

**Figure 6:**
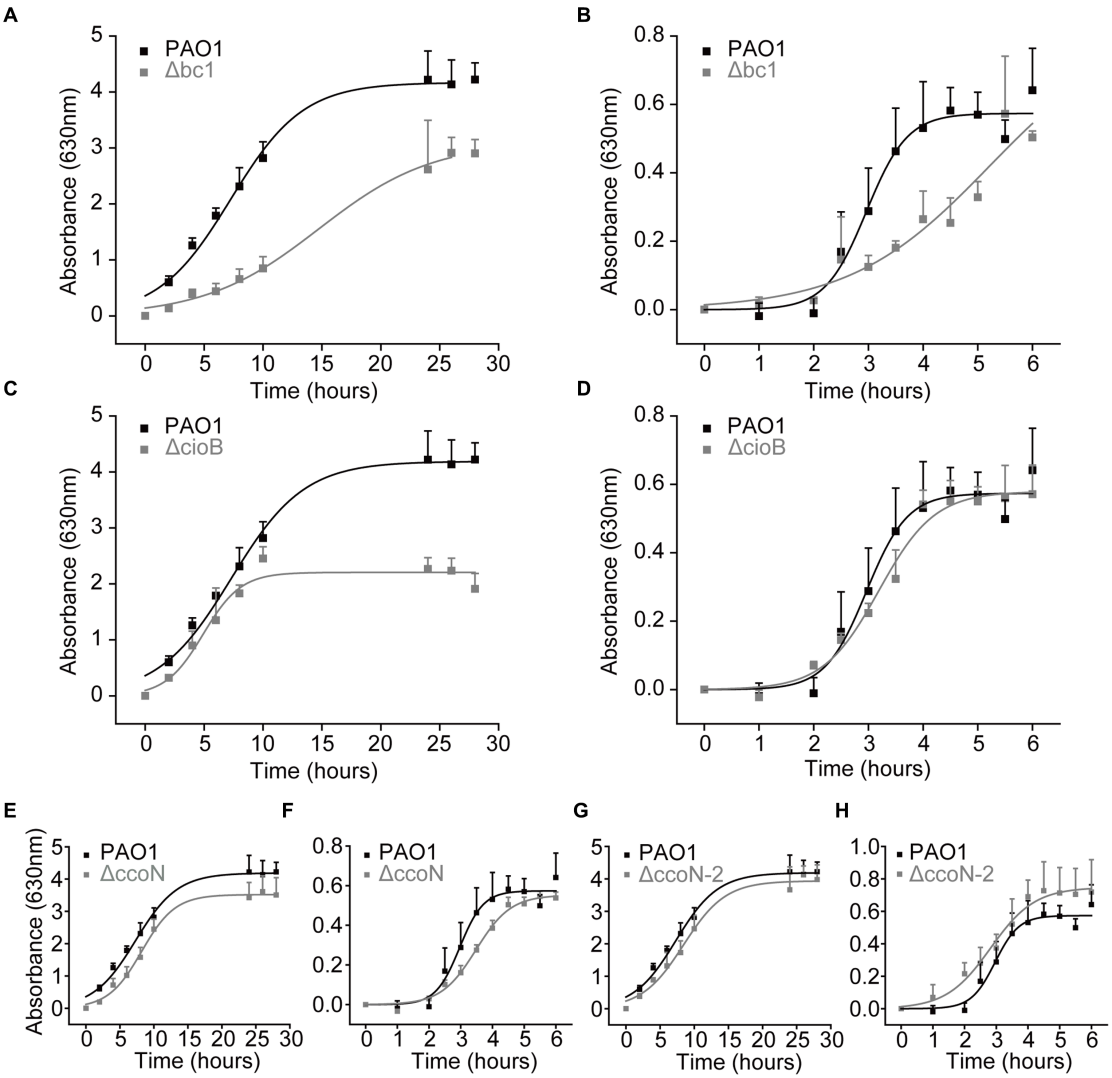
Growth of wild-type and mutant *P. aeruginosa* cells in LB and mAUM. *Pseudomonas aeruginosa* cells were grown in LB **(A,C,E,G)** and mAUM **(B,D,F,H)**. The growth of wild-type cells (black line) is compared to the mutants (gray line) Δbc1 (A and B), ΔcioB **(C,D)**, ΔccoN-1 **(E,F)** and ΔccoN-2 **(G,H)**. Data represented as mean and SD.

To corroborate the results of the functional analysis of the respiratory chain, we carried out an examination of the growth of mutants lacking genes of the respiratory complexes, using the PA14 library. The genes encoding the enzymes for the bc1 complex, cioB, ccoN-1, and ccoN-2 are identical between the PA14 and PAO1 strains, according to the PA14 Transposon Insertion Mutants Library,^3^ which provides the comparison data for each gene between the mutant of PA14 strain and its corresponding homologous gene in PAO1. A protein BLAST also reveals 100% identity for these enzymes in PAO1 and PA14 strains. As previously reported by our group (Liang et al., 2020) and others (Torres et al., 2019), the mutants of single NADH dehydrogenases do not produce noticeable effects on the growth in LB or mAUM, indicating that in the absence of any of them, the other two NADH dehydrogenases can compensate and rescue the growth. In contrast, the mutants of complex III (Δ*bc1*) and CIO oxidase (Δ*cioB*) show significant defects in the growth. The growth of the Δ*bc_1_* mutant in LB and mAUM shows a 2-fold decrease in the growth rate and a modest decrease in the yield in LB media (Figures 6A,B), which indicates that complex III plays significant roles in both media. The growth of the Δ*cioB* mutant in LB showed a reduction in the yield (Figure 6C), which is consistent with the large role that this enzyme plays in the stationary phase in this medium, but noticeable effects were not found in mAUM (Figure 6D). The *cbb*_3_-1 (ΔccoN) and *cbb*_3_-2 (ΔccoN-2) mutants do not show significant changes in the growth profile (Figures 6E–H).

## 4 Discussion

*Pseudomonas aeruginosa* is one of the main causal agents of multidrug-resistant infections and CAUTIs in the world, representing a significant health problem (Mittal et al., 2009; Weiner-Lastinger et al., 2020). *Pseudomonas aeruginosa* has a highly adaptive metabolism that allows it to survive in different environments, which likely contributes to the evolution of antibiotic resistance. In order to obtain new therapeutic treatments against *P. aeruginosa* UTIs and CAUTIs, it is necessary to understand its metabolic adaptations, in particular the roles played by the aerobic respiratory chain, of this notorious multi-drug resistant and ubiquitous pathogen in urine-like media. Previous studies demonstrate that the urine environment is aerobic, with oxygen’s concentration close to 100 μM, even in patients with urinary tract infections (Giannakopoulos et al., 1997), indicating that the aerobic respiratory chain has an important role in the physiology of this bacterium. Our previous characterization of the respiratory chain showed a very clear picture of the respiratory enzymes used by *P. aeruginosa* in the actively multiplying state. Moreover, our results clearly showed that transcriptomic and mutant studies must be supplemented with functional studies, as the results of gene expression profiles and mutant growth do not necessarily correlate with the functional architecture of the pathway (Liang et al., 2020). In this study, we have characterized the respiratory chain of *P. aeruginosa* in the stationary phase of growth in urine-like media, to understand the metabolism of this pathogen when CAUTIs or UTIs have been established. The stationary phase is particularly relevant in the clinic, as non-replicating cells might resemble the physiological state of cells in the urinary tract, as bacterial growth is prevented by the chemical composition of urine itself (Chambers et al., 1999; Bono et al., 2022) and by antibacterial coatings on the catheter (Andersen and Flores-Mireles, 2020). Moreover, long-term infection by this pathogen often involves plateauing in bacterial growth and biofilm formation (Trautner and Darouiche, 2004).

### 4.1 Metabolic activation in AUM

Urine is a complex fluid which could composed of more than 3,000 substance and varies among individuals and makes the study of an *in vitro* experimental condition difficult (Sarigul et al., 2019), especially because urine-like media is prone to salt precipitation (Mittal et al., 2009) and to drastic changes in pH, due to the chemical decomposition of urea (Levinsky and Berliner, 1959). AUM is made with the major components of human urine to generate a practical and reproducible formulation. In our previous study, we showed that mAUM, which was developed in our group, offers higher stability and reproducibility compared to other urine-like published media (Liang et al., 2020), and thus was selected to study and understand the physiology of bacteria in the urine.

Urine contains low nutrients, which limits energy production, explaining the reduced growth compared to the nutrient rich condition (Liang et al., 2020). Proteomics data show that *P. aeruginosa* has a very active aerobic metabolism and uses essentially the same pathways in LB and in mAUM media: the Entner-Duodoroff pathway, the pentose phosphate pathway, the Krebs cycle/ glyoxylate shunt and oxidative phosphorylation. Even though all respiratory enzymes were found as core proteins, present in all conditions evaluated, the regulation of the activity of some of these enzymes is tightly controlled by the cell and several of them are activated in urine-like media. Our data show that the respiratory activity of stationary phase cells grown in mAUM exhibits a significant increase of approximately 3 times compared to the activity in the logarithmic phase. In contrast with the expectation that the cells might become quiescent and perhaps decrease their metabolic rate due to the lack of nutrients, which is observed at a low extent in LB media (Figure 3), we noticed that in urine-like media the activity of all respiratory enzymes is higher. This metabolic activation could be related to the need to maximize the energy production from the limited resources available, in particular from lactate which is one of the main components of urine (Scheijen et al., 2012). Although major metabolic changes were found, our qPCR results on NQR did not point to changes in the transcriptional activity, indicating that other levels of protein or enzymatic regulations are involved in the respiratory activation of the cells.

### 4.2 Role of NADH dehydrogenases

In this study, we are showing that the NADH-dependent oxygen consumption rate increases significantly in the stationary phase in cells grown in mAUM. Through a functional characterization of the respiratory activity, we were able to determine that NQR is the main NADH dehydrogenase in all conditions tested, with a minor role by complex I and a negligible participation of NDH-2. These results are similar to the behavior that we have reported previously in the logarithmic phase of growth (Liang et al., 2020), which also show that NQR is the main NADH dehydrogenase. The reasons behind this metabolic strategy have been unclear until now, and we can start to elucidate the advantages of this pathway architecture. It is likely that NDH-2 plays a minor role in the respiratory activity since is not electrogenic and it is not coupled to the synthesis of ATP (Kerscher et al., 2008; Heikal et al., 2014). On the other hand, complex I and NQR pump protons with different stoichiometries, of 2 (Wikström and Hummer, 2012) and 1 H^+^ (Bogachev et al., 1997) per electron delivered to ubiquinone, respectively. While complex I appears to be more energy efficient, there are several advantages of NQR that could explain why *P. aeruginosa* uses it as the main NADH dehydrogenase. For instance, complex I contains two 2Fe-2S and six 4Fe-2S clusters (Yagi, 1991; Yagi et al., 1998; Kerscher et al., 2008), while NQR contains only two 2Fe-2S centers (Kishikawa et al., 2022). The requirement of minimum iron use of NQR supports the major role that it plays in pathogens (Reyes-Prieto et al., 2014), as iron is normally a limiting factor in pathogen’s growth (Cassat and Skaar, 2013). Moreover, the molecular weight of NQR is only half compared to complex I, which would require less energy and faster assembly. In addition, complex I is one of the main sources of reactive oxygen species (McLennan and Degli Esposti, 2000). While the rates of NADH oxidation and ubiquinone reduction are similar in NQR (Juárez et al., 2009), indicating that most electrons are delivered to the physiological substrate, in complex I the oxidation of NADH is 20–30 times higher compared to ubiquinone reduction (Fedor et al., 2017), which would produce significantly more oxygen radicals, and also would decrease the effective energy yield of the complex. Another important advantage of NQR is related to the resistance against self-poisoning. HQNO is a molecule produced by *P. aeruginosa* for quorum sensing and biofilm formation, which eliminates competing bacteria by inhibiting their respiratory activity (Déziel et al., 2004; Raba et al., 2018). Our previous study shows that *P. aeruginosa* NQR is only partially sensitive to HQNO inhibition, which allows the bacteria to survive autopoisoning and outcompete other bacteria (Raba et al., 2019). Even though NQR is the preferred NADH dehydrogenase by the bacteria, our previous studies (Liang et al., 2020) and other reports (Torres et al., 2019) indicate that its genetic inactivation does not produce significant changes in the growth parameters, which is puzzling considering the major role that it plays. However, by analyzing the contribution of all respiratory chain enzymes we can start to understand this and other phenotypes (see below).

### 4.3 Role of terminal oxidases

The role of aerobic terminal oxidases was also studied, as they should play a major part in the aerobic urinary medium. In LB medium, there is a major change in the metabolic strategy used between the two phases of growth, from the use of CYO as the main oxidase in the logarithmic phase, to the use of CIO in the stationary phase. In rich media, *P. aeruginosa* as well as many other bacteria such as *P. putida*, *Escherichia coli*, and *Rhizobium etli* (Iuchi et al., 1990; Ugidos et al., 2008; Lunak and Noel, 2015) use CYO oxidases, which could be a response to high oxygen concentration in the initial part of growth, as described before (Liang et al., 2020). As the cell growth rate decreases, the concentration of oxygen might decrease, and the cells start producing cyanide (Cody et al., 2009), which can reach up to 500 μM (Lenney and Gilchrist, 2011), triggering the switch to the cyanide-resistant CIO oxidase that allows the continuous operation of the electron transfer chain, supporting ATP synthesis (Kawakami et al., 2010; Yan et al., 2019). On the other hand, in mAUM the cells maintain their metabolic profile, with complex III and COX oxidases participating with 50–60% of the electron flow. However, an increase in CIO activity in the stationary phase (10% approximately) was noticeable, which could be related to an increase in cyanide production. Previous studies indicate that *P. aeruginosa* adapts its metabolic machinery to optimize the energy yield and survive environmental changes, especially in nutrient barren conditions (Frimmersdorf et al., 2010), such as the urine. Because of the constraints of the mAUM components, ATP yield is likely to be a major factor influencing the metabolic strategy utilized. The use of the pathway composed of complex III and COXs appears as optimal, as these enzymes pump 6 (4 and 2, respectively) protons per oxygen atom reduced (Brand, 1994), while both CYO (Graf et al., 2019) and CIO (D’mello et al., 1996) oxidases only pump four and two protons per oxygen reduced, respectively.

In contrast with the behavior of the NADH dehydrogenase mutants. The growth is significantly affected in mutants of the terminal oxidases. For instance, our data show that the CIO mutant is not affected in mAUM, consistent with its minor role in this condition, and shows a decrease in the yield in LB media, reflecting its importance in the stationary phase of growth. On the other hand, the growth rate of the of the Δ*bc*_1_ mutant was significantly reduced in mAUM, consistent with the main role that we are describing above. Interestingly, this mutant also shows significant changes in the growth rate in LB media, even though it seems to have a small role compared to other oxidases in this condition. Our results also show that the mutants of *cbb*_3_ oxidases do not produce a significant decrease in the growth, indicating that the two other COX oxidases can compensate the lack of any of the other two, but also highlighting the role of complex III in energy generation. These studies were performed with the more virulent strain PA14, carrying two pathogenicity islands that are absent in PAO1, due to accessibility of mutants. However, the genomes of PAO1 and PA14 strains are very similar (Mikkelsen et al., 2011) and the genes and protein products for all the respiratory complexes mutants that we tested, are identical between the two strains. Therefore, we expect a similar growth behavior for both strains. Moreover, genome-wide genetic studies performed for *P. aeruginosa* PA14 and several clinical isolates grown in different conditions (including urine) reveal that the essential core genes across the isolates and growth conditions include those that encode for the respiratory enzymes described in this study. Thus, different metabolic strategies are not expected among strains (Poulsen et al., 2019).

### 4.4 Contribution of the respiratory complexes to *Pseudomonas aeruginosa* energy metabolism

**Figure 7:**
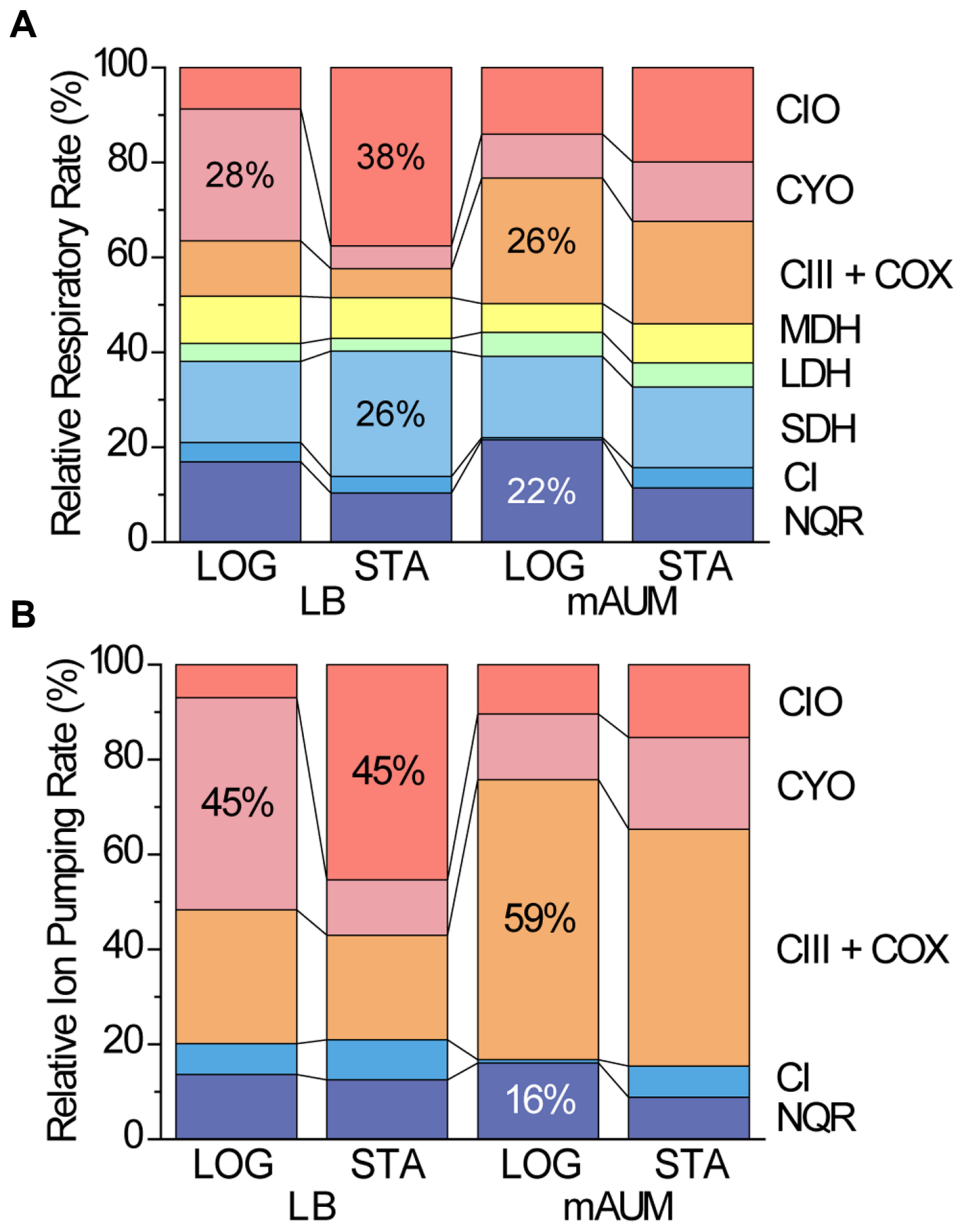
Relative contribution of respiratory enzymes to the respiratory rate **(A)** and to the ion pumping rate of *P. aeruginosa* cells grown in different conditions **(B)**. **(A)** Normalized respiratory rate of major respiratory enzymes and **(B)** normalized ion pumping rate of respiratory enzymes in logarithmic (LOG) and stationary (STA) phases of cells grown in LB and mAUM.

In order to understand the role that each of the respiratory complexes play in the energy metabolism of *P. aeruginosa*, we carried out an analysis of their relative contribution to the respiratory activity and to ATP synthesis (Figure 7). To estimate the contribution to the respiratory activity we assumed that the dehydrogenases activities are rate limiting, since the terminal oxidases appear to be much more active in comparison (Figure 3). We also assumed that the physiologic concentrations of their substrates are close to the concentrations used in our experiments, and thus they all can participate with rates close to those observed individually. Finally, we assumed that substrate channeling between individual complexes, as well as microdomains, which might divert preferentially electron flow to some complexes, is negligible. With this information and considering the relative participation of NADH dehydrogenases and terminal oxidases to the respiratory rate, we were able to estimate the relative contribution of all complexes to the respiratory metabolism (Supplementary Tables S1, S2). The data obtained are presented in Figure 7A, showing that the main dehydrogenases in all conditions are NQR and SDH, carrying out about 40% of all the respiratory activity. Depending on the media, the main oxidases are CYO, CIO, or complex III plus COX oxidases. To fully understand the role of these enzymes in energy metabolism, we also estimated their roles in the generation of ATP, using the known ion pumping (H^+^/e-) stoichiometry for NQR [1, (Bogachev et al., 1997)], Complex I [2, (Wikström and Hummer, 2012)], Complex III and COX [4 and 2, (Brand, 1994)], CYO [4, (Graf et al., 2019)] and CIO [3, (D’mello et al., 1996)]. We would like to mention that other stoichiometries have been reported for *P. aeruginosa* respiratory complexes (Arai et al., 2014), but the ratios reported do not consider the contribution of NADH dehydrogenases and thus are likely overestimated. Figure 7B, shows the relative contributions of all measured respiratory enzymes to the proton pumping activity. In all conditions tested, the vast majority (>80%) of the ATP synthesis is carried out by the terminal oxidases, with NQR being the most important dehydrogenase, but playing a comparatively smaller role. These results allow us to understand the lack of effects of NQR elimination on the growth of the bacteria, as its contribution can be compensated by other enzymes, not only by the NADH dehydrogenases. The data also show that complex III and COX oxidases play significant roles in all conditions (>20%), but especially in mAUM, with a 50% participation to the overall ATP production. This effect is very well reflected in the phenotype of the Δ*bc*_1_ mutant, showing important decreases in the growth rate and yield, which can be compensated, but only partially by other oxidases. This analysis finally provides a framework to understand the metabolism used by this important pathogen, which has remained unknown despite several decades of work by different groups.

### 4.5 Metabolism guided antibiotic design

The results of this study provide a way to understand the role of respiratory enzymes in the metabolism of *P. aeruginosa*. Understanding the significance of the aerobic respiration is crucial as they play a pivotal role in maintaining bacterial growth, particularly in the context of urinary tract infections. In particular, we are describing that Complex III, NQR and SDH play important roles in cell physiology and may be targeted with new antibiotics. Complex III appears as a suitable target, as previous reports show that antiparasitic drugs targeting the microbial complex III can be obtained (Stickles et al., 2015). NQR also plays an important role, as the main NADH dehydrogenase, which could be targeted by inhibitors, as recently described for *Chlamydia trachomatis* (Dibrov et al., 2017a; Liang et al., 2018). However, our data shows that the elimination of the NQR gene does not produce significant effects on the growth of the bacteria. Nonetheless, it is likely that its disruption can produce a dysregulation of the metabolism and affect pathogenicity. Indeed, it has been shown that NQR deletion in *V. cholerae* decreases the expression of the cholera toxin (Minato et al., 2014). Finally, SDH is another potential target that could be used to design new drugs. Indeed, a recent report (Mogi et al., 2009) has shown that the inhibitor siccanin is able to specifically inhibit *P. aeruginosa* SDH, with no significant toxicity to human cells. Further characterizations are required to identify and validate these and other pharmacologic targets, which are urgently needed to treat CAUTIs and multidrug resistant infections by this microorganism.

## Data availability statement

The Mass spectrometry data presented in the study are deposited in the MassIVE repository, accession number PXD047660. http://massive.ucsd.edu/ProteoSAFe/dataset.jsp?task=7184a7a931fe4a08b7f4bdb03c4cbc84.

## Author contributions

YH: Formal analysis, Investigation, Validation, Visualization, Writing – original draft, Writing – review & editing. MY: Investigation, Validation, Visualization, Writing – original draft. AJ: Formal analysis, Investigation, Software, Writing – original draft. KT: Conceptualization, Data curation, Formal analysis, Funding acquisition, Investigation, Methodology, Project administration, Resources, Software, Supervision, Validation, Visualization, Writing – original draft, Writing – review & editing. OJ: Conceptualization, Data curation, Formal analysis, Funding acquisition, Investigation, Methodology, Project administration, Resources, Software, Supervision, Validation, Visualization, Writing – original draft, Writing – review & editing.

## Footnotes

^1^https://www.kegg.jp/kegg/catalog/org_list.html

^2^https://github.com/AlexanderThomasJulian/MassSpec2KEGG

^3^https://pa14.mgh.harvard.edu/cgi-bin/pa14/home.cgi
