# Supplementary material for: Identification of complex III, NQR, and SDH as primary bioenergetic enzymes during the stationary phase of Pseudomonas aeruginosa cultured in urine-like conditions
Source: Front Microbiol. 2024 Feb 21;15:1347466. doi: 10.3389/fmicb.2024.1347466 (PMC10926992; doi:10.3389/fmicb.2024.1347466)
Supplement: Supplementary file 3 [file Data_Sheet_1.PDF]

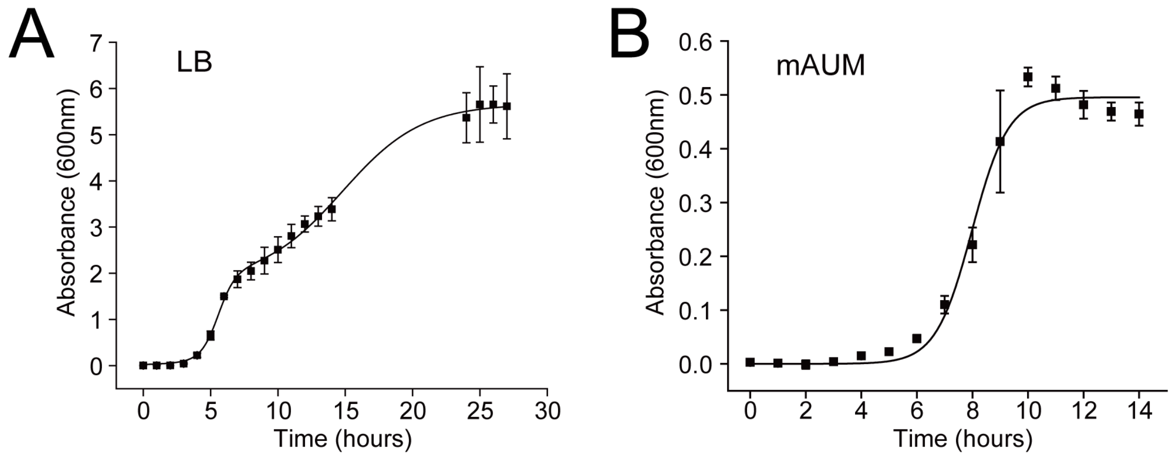

Supplementary Figure 1. Growth of *P. aeruginosa* PAO1 in LB (A) and mAUM (B). The growth curves were fitted using a logistic function (Kahm *et al.*, 2010). Data are expressed as mean ± SD.
