# Supplementary material for: Identification of complex III, NQR, and SDH as primary bioenergetic enzymes during the stationary phase of Pseudomonas aeruginosa cultured in urine-like conditions
Source: Front Microbiol. 2024 Feb 21;15:1347466. doi: 10.3389/fmicb.2024.1347466 (PMC10926992; doi:10.3389/fmicb.2024.1347466)
Supplement: Supplementary file 4 [file Data_Sheet_2.PDF]

Supplementary Tables

Table S1. Normalized respiratory rate of major respiratory enzymes in logarithmic (LOG) and stationary (STA) phases of cells grown in LB and mAUM.

Enzyme (%)	LB		mAUM	
	LOG	STA	LOG	STA
NQR	16.9	10.3	21.6	11.4
Complex I	4.1	3.5	0.5	4.3
SDH	17.1	26.4	17.1	17.0
LDH	3.8	2.7	5.1	5.1
MDH	9.9	8.6	6.1	8.2
COX	11.7	6.1	26.4	21.5
CYO	27.8	4.8	9.3	12.5
CIO	8.7	37.6	14.0	19.9

Table S2. Normalized ion pumping rate of respiratory enzymes in logarithmic (LOG) and stationary (STA) phases of cells grown in LB and mAUM.

Enzyme (%)	LB		mAUM	
	LOG	STA	LOG	STA
NQR	13.6	12.5	16.0	8.8
Complex I	6.5	8.4	0.7	6.6
COX	28.2	22.0	59.0	49.9
CYO	44.7	11.6	13.8	19.3
CIO	7.0	45.4	10.4	15.4
